# Dimethyl 3,3′-diphenyl-2,2′-[(*S*)-thio­phene-2,5-diylbis(carbonyl­aza­nedi­yl)]dipropano­ate tetra­hydro­furan monosolvate

**DOI:** 10.1107/S1600536810034410

**Published:** 2010-09-04

**Authors:** GuangMing Xia, Jing Liu, Zhen Li, MuWei Ji, GuoXin Sun

**Affiliations:** a Shandong Provincial Key Laboratory of Fluorine Chemistry and Chemical Materials, School of Chemistry and Chemical Engineering, University of Jinan, Ji’nan 250022, People’s Republic of China; b School of Chemistry and Chemical Engineering, University of Jinan, Ji’nan 250022, People’s Republic of China

## Abstract

The title compound, C_26_H_26_N_2_O_6_S·C_4_H_8_O, a solvated bis-amide derivative, is also a chiral amino acid ester with l-phenyl­alanine methyl ester groups as amine substituents. The thio­phene-2,5-dicarboxamide core approximates *C*_2_ point symmetry. The tetra­hydro­furan solvent mol­ecule is linked to the main mol­ecule through an inter­molecular N—H⋯O hydrogen bond. The central ring makes dihedral angles of 90.0 (2) and 76.5 (2)° with the pendant rings.

## Related literature

For applications of thio­phene derivatives, see: Zhao *et al.* (2009 ▶). For the synthesis of the title compound, see: Moriuchi *et al.* (2006 ▶). For the structure of the unsolvated mol­ecule, see: Xia *et al.* (2010 ▶).
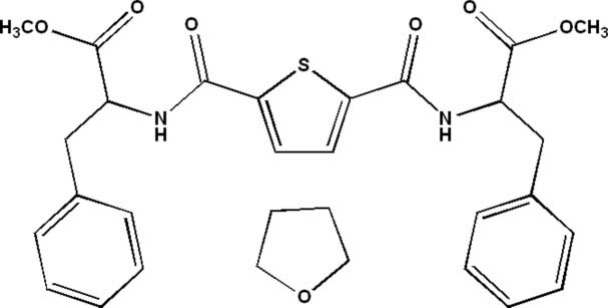


## Experimental

### 

#### Crystal data

C_26_H_26_N_2_O_6_S·C_4_H_8_O
*M* *_r_* = 566.65
Orthorhombic, 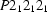

*a* = 8.3041 (3) Å
*b* = 12.1810 (4) Å
*c* = 29.6787 (11) Å
*V* = 3002.06 (17) Å^3^
*Z* = 4
Mo *K*α radiation
μ = 0.16 mm^−1^
*T* = 293 K
0.40 × 0.21 × 0.08 mm

#### Data collection

Oxford Xcalibur (Eos) CCD detector diffractometer
Absorption correction: multi-scan (*CrysAlis PRO RED*; Oxford Diffraction, 2009 ▶) *T*_min_ = 0.941, *T*_max_ = 0.988
7782 measured reflections
5081 independent reflections
3167 reflections with *I* > 2σ(*I*)
*R*_int_ = 0.022

#### Refinement

*R*[*F* ^2^ > 2σ(*F* ^2^)] = 0.047
*wR*(*F* ^2^) = 0.094
*S* = 0.91
5081 reflections
363 parameters
H-atom parameters constrained
Δρ_max_ = 0.19 e Å^−3^
Δρ_min_ = −0.21 e Å^−3^
Absolute structure: Flack (1983 ▶), 1964 Friedel pairs
Flack parameter: −0.05 (9)

### 

Data collection: *CrysAlis PRO CCD* (Oxford Diffraction, 2009 ▶); cell refinement: *CrysAlis PRO CCD*; data reduction: *CrysAlis PRO RED* (Oxford Diffraction, 2009 ▶); program(s) used to solve structure: *SHELXS97* (Sheldrick, 2008 ▶); program(s) used to refine structure: *SHELXL97* (Sheldrick, 2008 ▶); molecular graphics: *Mercury* (Macrae *et al.*, 2008 ▶); software used to prepare material for publication: *SHELXL97*.

## Supplementary Material

Crystal structure: contains datablocks global, I. DOI: 10.1107/S1600536810034410/bh2306sup1.cif

Structure factors: contains datablocks I. DOI: 10.1107/S1600536810034410/bh2306Isup2.hkl

Additional supplementary materials:  crystallographic information; 3D view; checkCIF report

## Figures and Tables

**Table 1:** Hydrogen-bond geometry (Å, °)

*D*—H⋯*A*	*D*—H	H⋯*A*	*D*⋯*A*	*D*—H⋯*A*
N2—H2⋯O7	0.86	2.02	2.859 (3)	164

**Fig. 1.**
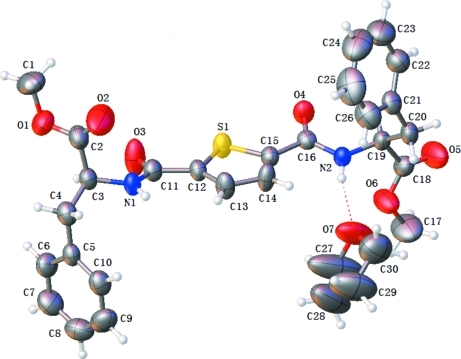
The title structure with thermal ellipsoids at 30% probability level.

## supplementary crystallographic information

### Comment

The thiophene derivatives have been viewed as significant compounds for applications in many fields, such as photo-materials, electronic luminescence materials (Zhao *et al.*, 2009). The title compound derives from thiophene-2,5-dicarboxylic acid and a natural amino acid. This makes this kind of structures very promising for biological activities and as precursors in the synthesis of various compounds.

In the structure of the title compound, the thiophene-2,5-dicarboxamide core approximates *C*_2_ point symmetry. The molecules are connected by intermolecular N—H···O hydrogen-bonding interactions with tetrahydrofuran molecules. Chiral atoms C3 and C19 in the main molecule retain the absolute *S* configuration of the parent *L*-phenylalanine. The procedure used for the synthesis of the title compound is thus carried-out without inversion for chiral centers. The structure of the unsolvated molecule with identical absolute configuration has been determined (Xia *et al.*, 2010).

### Experimental

A mixture of 2,5-thiophenedicarboxylic acid (0.3 mmol), thionyl chloride (3 mmol) and 3–5 drops of *N*,*N*-dimethylformamide in a flask was heated to 343 K for 10 h. The resulting solution was evaporated under vacuum, affording 2,5-thiophenedicarbonyldichloride as a pale yellow product.

The title compound was synthesized by a slight modification of a procedure described by Moriuchi *et al.* (2006). To a stirred mixture of *L*-phenylalanine methyl ester hydrochloride (129.4 mg, 0.6 mmol in 15 ml of dry dichloromethane) and triethylamine (0.21 ml, 1.5 mmol) was added dropwise 2,5-thiophenedicarbonyldichloride (62.7 mg, 0.3 mmol) in dichloromethane (3 ml) at 253 K and then 20 h at 293 K. The resulting mixture was diluted with dichloromethane, washed with saturated NaHCO_3_ solution and brine, and then dried over anhydrous MgSO_4_. The solvent was removed *in vacuo*. The title compound was isolated as a white solid by crystallization from 2-propanol (yield: 129.6 mg, 78%). Then the product was recrystallized from THF, to yield colourless blocks of the title solvate.

### Refinement

All H atoms were placed in idealized positions and refined using a riding model, with N—H = 0.86 Å, and C—H = 0.93–0.98 Å, and with *U*_iso_(H) = 1.2–1.5*U*_eq_(carrier atom). Absolute configuration was assigned by refinement of the Flack parameter (Flack, 1983), based on 1964 measured Friedel pairs.

### Crystal data

**Table:**

C_26_H_26_N_2_O_6_S·C_4_H_8_O	*F*(000) = 1200
*M**_r_* = 566.65	*D*_x_ = 1.254 Mg m^−^^3^
Orthorhombic, *P*2_1_2_1_2_1_	Mo *K*α radiation, λ = 0.71073 Å
Hall symbol: P 2ac 2ab	Cell parameters from 3062 reflections
*a* = 8.3041 (3) Å	θ = 3.0–28.8°
*b* = 12.1810 (4) Å	µ = 0.16 mm^−^^1^
*c* = 29.6787 (11) Å	*T* = 293 K
*V* = 3002.06 (17) Å^3^	Block, colourless
*Z* = 4	0.40 × 0.21 × 0.08 mm

### Data collection

**Table:**

Oxford Xcalibur (Eos) CCD detector diffractometer	5081 independent reflections
Radiation source: Enhance (Mo) X-ray Source	3167 reflections with *I* > 2σ(*I*)
graphite	*R*_int_ = 0.022
Detector resolution: 16.0355 pixels mm^-1^	θ_max_ = 25.3°, θ_min_ = 3.0°
ω scans	*h* = −9→9
Absorption correction: multi-scan (*CrysAlis PRO* RED; Oxford Diffraction, 2009)	*k* = −14→12
*T*_min_ = 0.941, *T*_max_ = 0.988	*l* = −15→35
7782 measured reflections	

### Refinement

**Table:**

Refinement on *F*^2^	Secondary atom site location: difference Fourier map
Least-squares matrix: full	Hydrogen site location: inferred from neighbouring sites
*R*[*F*^2^ > 2σ(*F*^2^)] = 0.047	H-atom parameters constrained
*wR*(*F*^2^) = 0.094	*w* = 1/[σ^2^(*F*_o_^2^) + (0.0458*P*)^2^] where *P* = (*F*_o_^2^ + 2*F*_c_^2^)/3
*S* = 0.91	(Δ/σ)_max_ < 0.001
5081 reflections	Δρ_max_ = 0.19 e Å^−^^3^
363 parameters	Δρ_min_ = −0.20 e Å^−^^3^
0 restraints	Absolute structure: Flack (1983), 1964 Friedel pairs
0 constraints	Flack parameter: −0.05 (9)
Primary atom site location: structure-invariant direct methods	

### Fractional atomic coordinates and isotropic or equivalent isotropic displacement parameters (Å^2^)

**Table:**

	*x*	*y*	*z*	*U*_iso_*/*U*_eq_	
C1	0.7362 (4)	−0.1856 (4)	−0.38825 (15)	0.0993 (14)	
H1A	0.7425	−0.2168	−0.3586	0.149*	
H1B	0.7790	−0.1124	−0.3878	0.149*	
H1C	0.7976	−0.2297	−0.4088	0.149*	
C2	0.4695 (4)	−0.1250 (3)	−0.37793 (13)	0.0615 (9)	
C3	0.2978 (3)	−0.1273 (2)	−0.39603 (10)	0.0476 (8)	
H3	0.2785	−0.2003	−0.4089	0.057*	
C4	0.2776 (4)	−0.0426 (2)	−0.43389 (11)	0.0555 (8)	
H4A	0.2911	0.0305	−0.4215	0.067*	
H4B	0.3615	−0.0538	−0.4562	0.067*	
C5	0.1160 (4)	−0.0496 (2)	−0.45669 (10)	0.0466 (8)	
C6	0.0864 (4)	−0.1295 (3)	−0.48868 (12)	0.0638 (9)	
H6	0.1673	−0.1793	−0.4960	0.077*	
C7	−0.0602 (5)	−0.1369 (3)	−0.50983 (13)	0.0782 (11)	
H7	−0.0772	−0.1897	−0.5319	0.094*	
C8	−0.1798 (5)	−0.0671 (4)	−0.49844 (15)	0.0794 (12)	
H8	−0.2804	−0.0741	−0.5119	0.095*	
C9	−0.1543 (5)	0.0131 (3)	−0.46754 (16)	0.0804 (12)	
H9	−0.2361	0.0624	−0.4606	0.097*	
C10	−0.0064 (4)	0.0216 (2)	−0.44637 (11)	0.0628 (9)	
H10	0.0102	0.0761	−0.4249	0.075*	
C11	0.1316 (3)	−0.1966 (3)	−0.33570 (10)	0.0476 (7)	
C12	0.0384 (3)	−0.1773 (2)	−0.29456 (10)	0.0428 (7)	
C13	−0.0220 (4)	−0.0850 (2)	−0.27630 (10)	0.0588 (9)	
H13	−0.0123	−0.0160	−0.2894	0.071*	
C14	−0.1017 (4)	−0.1042 (2)	−0.23495 (12)	0.0609 (9)	
H14	−0.1489	−0.0490	−0.2178	0.073*	
C15	−0.1023 (3)	−0.2104 (2)	−0.22293 (9)	0.0425 (7)	
C16	−0.1644 (3)	−0.2650 (2)	−0.18143 (10)	0.0410 (7)	
C17	−0.7408 (4)	−0.3073 (4)	−0.15049 (14)	0.1109 (16)	
H17A	−0.8026	−0.2648	−0.1294	0.166*	
H17B	−0.7452	−0.3834	−0.1422	0.166*	
H17C	−0.7846	−0.2983	−0.1802	0.166*	
C18	−0.4957 (4)	−0.2807 (2)	−0.11222 (12)	0.0541 (8)	
C19	−0.3256 (3)	−0.2374 (2)	−0.11366 (10)	0.0449 (7)	
H19	−0.2553	−0.2970	−0.1034	0.054*	
C20	−0.3044 (3)	−0.1424 (2)	−0.08026 (11)	0.0543 (9)	
H20B	−0.3664	−0.0799	−0.0906	0.065*	
H20A	−0.3466	−0.1643	−0.0511	0.065*	
C21	−0.1327 (4)	−0.1090 (3)	−0.07497 (11)	0.0532 (9)	
C22	−0.0324 (4)	−0.1616 (3)	−0.04495 (12)	0.0687 (10)	
H22	−0.0732	−0.2182	−0.0273	0.082*	
C23	0.1266 (6)	−0.1322 (4)	−0.04062 (16)	0.0960 (14)	
H23	0.1916	−0.1688	−0.0200	0.115*	
C24	0.1900 (6)	−0.0506 (5)	−0.0661 (2)	0.1118 (18)	
H24	0.2977	−0.0309	−0.0634	0.134*	
C25	0.0910 (7)	0.0024 (4)	−0.0962 (2)	0.1108 (16)	
H25	0.1327	0.0587	−0.1139	0.133*	
C26	−0.0682 (5)	−0.0260 (3)	−0.10075 (14)	0.0785 (11)	
H26	−0.1327	0.0111	−0.1214	0.094*	
C27	−0.5480 (9)	−0.0480 (5)	−0.2287 (2)	0.210 (4)	
H27A	−0.4867	−0.0942	−0.2490	0.252*	
H27B	−0.6468	−0.0857	−0.2207	0.252*	
C28	−0.5824 (9)	0.0546 (6)	−0.2491 (2)	0.184 (3)	
H28B	−0.6961	0.0597	−0.2563	0.221*	
H28A	−0.5211	0.0629	−0.2767	0.221*	
C29	−0.5388 (8)	0.1382 (5)	−0.2175 (2)	0.170 (3)	
H29B	−0.6284	0.1875	−0.2121	0.204*	
H29A	−0.4480	0.1806	−0.2285	0.204*	
C30	−0.4949 (7)	0.0780 (4)	−0.17561 (19)	0.1219 (16)	
H30A	−0.4048	0.1133	−0.1607	0.146*	
H30B	−0.5854	0.0758	−0.1550	0.146*	
N1	0.1852 (3)	−0.11168 (19)	−0.35934 (8)	0.0479 (6)	
H1	0.1527	−0.0466	−0.3528	0.057*	
N2	−0.2705 (2)	−0.20642 (19)	−0.15812 (8)	0.0430 (6)	
H2	−0.3083	−0.1474	−0.1700	0.052*	
O1	0.5682 (3)	−0.1824 (2)	−0.40280 (8)	0.0745 (7)	
O2	0.5104 (3)	−0.0779 (3)	−0.34523 (11)	0.1289 (13)	
O3	0.1599 (3)	−0.29143 (18)	−0.34747 (8)	0.0910 (8)	
O4	−0.1172 (2)	−0.35693 (15)	−0.17022 (7)	0.0555 (6)	
O5	−0.5526 (3)	−0.3183 (3)	−0.07888 (10)	0.1001 (9)	
O6	−0.5731 (3)	−0.2699 (2)	−0.15000 (9)	0.0818 (8)	
O7	−0.4551 (4)	−0.0230 (2)	−0.18881 (12)	0.1181 (11)	
S1	−0.00131 (10)	−0.28933 (5)	−0.26159 (3)	0.0543 (2)	

### Atomic displacement parameters (Å^2^)

**Table:**

	*U*^11^	*U*^22^	*U*^33^	*U*^12^	*U*^13^	*U*^23^
C1	0.054 (2)	0.142 (4)	0.102 (3)	0.014 (2)	0.001 (2)	0.028 (3)
C2	0.060 (2)	0.079 (2)	0.045 (2)	−0.0056 (19)	0.004 (2)	−0.0055 (19)
C3	0.0561 (19)	0.0494 (17)	0.037 (2)	−0.0002 (15)	0.0048 (17)	−0.0052 (16)
C4	0.064 (2)	0.0541 (19)	0.048 (2)	−0.0067 (16)	0.0060 (19)	−0.0004 (17)
C5	0.0574 (18)	0.0435 (17)	0.039 (2)	−0.0019 (16)	0.0095 (17)	0.0004 (16)
C6	0.073 (2)	0.062 (2)	0.056 (2)	0.0068 (18)	0.002 (2)	−0.015 (2)
C7	0.090 (3)	0.081 (3)	0.064 (3)	−0.006 (2)	−0.011 (2)	−0.014 (2)
C8	0.077 (3)	0.083 (3)	0.079 (3)	−0.003 (2)	−0.019 (3)	0.016 (3)
C9	0.075 (3)	0.073 (3)	0.093 (4)	0.020 (2)	0.006 (3)	0.020 (3)
C10	0.080 (2)	0.0543 (18)	0.054 (2)	0.001 (2)	0.004 (2)	−0.0020 (16)
C11	0.0565 (17)	0.0441 (18)	0.042 (2)	0.0000 (16)	0.0048 (16)	−0.0058 (16)
C12	0.0481 (16)	0.0448 (16)	0.0357 (18)	−0.0063 (13)	0.0065 (15)	−0.0025 (14)
C13	0.083 (2)	0.0396 (16)	0.053 (2)	−0.0001 (17)	0.022 (2)	0.0063 (15)
C14	0.078 (2)	0.0443 (18)	0.061 (2)	0.0057 (15)	0.032 (2)	−0.0004 (17)
C15	0.0430 (14)	0.0451 (17)	0.0396 (19)	−0.0047 (13)	0.0040 (14)	−0.0019 (15)
C16	0.0402 (16)	0.0413 (17)	0.042 (2)	−0.0075 (13)	−0.0025 (15)	−0.0023 (15)
C17	0.058 (2)	0.169 (4)	0.106 (4)	−0.041 (3)	−0.019 (2)	0.003 (3)
C18	0.0449 (17)	0.0638 (18)	0.053 (2)	0.0000 (18)	0.006 (2)	0.0113 (18)
C19	0.0463 (17)	0.0491 (17)	0.0394 (19)	0.0077 (14)	0.0069 (16)	0.0049 (15)
C20	0.059 (2)	0.064 (2)	0.041 (2)	0.0049 (16)	0.0100 (17)	−0.0013 (17)
C21	0.063 (2)	0.052 (2)	0.044 (2)	−0.0059 (17)	0.0036 (19)	−0.0103 (17)
C22	0.070 (3)	0.075 (2)	0.062 (3)	0.0000 (19)	0.000 (2)	0.004 (2)
C23	0.080 (3)	0.123 (4)	0.085 (4)	−0.001 (3)	−0.019 (3)	−0.014 (3)
C24	0.070 (3)	0.142 (5)	0.123 (5)	−0.022 (3)	−0.009 (3)	−0.044 (4)
C25	0.111 (4)	0.107 (4)	0.115 (5)	−0.058 (3)	0.026 (4)	−0.003 (3)
C26	0.090 (3)	0.070 (2)	0.076 (3)	−0.021 (2)	0.004 (2)	0.009 (2)
C27	0.296 (9)	0.128 (4)	0.205 (8)	0.092 (5)	−0.165 (8)	−0.053 (5)
C28	0.273 (8)	0.179 (6)	0.100 (5)	0.115 (6)	−0.059 (5)	−0.021 (5)
C29	0.221 (7)	0.108 (4)	0.183 (7)	0.041 (4)	−0.082 (6)	0.015 (5)
C30	0.136 (4)	0.105 (3)	0.125 (4)	0.033 (3)	−0.008 (4)	−0.011 (3)
N1	0.0621 (15)	0.0417 (14)	0.0399 (17)	0.0044 (12)	0.0133 (14)	−0.0017 (12)
N2	0.0471 (13)	0.0418 (12)	0.0401 (15)	0.0052 (12)	0.0075 (12)	0.0059 (13)
O1	0.0563 (14)	0.0945 (17)	0.0728 (18)	0.0055 (13)	0.0091 (13)	−0.0053 (15)
O2	0.0844 (19)	0.212 (3)	0.090 (2)	0.004 (2)	−0.0221 (19)	−0.072 (2)
O3	0.141 (2)	0.0481 (14)	0.0839 (19)	−0.0028 (15)	0.0556 (17)	−0.0047 (14)
O4	0.0709 (13)	0.0386 (11)	0.0568 (16)	0.0086 (10)	0.0112 (12)	0.0071 (11)
O5	0.0672 (16)	0.151 (2)	0.082 (2)	−0.0221 (15)	0.0101 (14)	0.047 (2)
O6	0.0554 (13)	0.128 (2)	0.0623 (17)	−0.0274 (13)	−0.0090 (13)	0.0150 (16)
O7	0.161 (3)	0.0830 (19)	0.110 (3)	0.054 (2)	−0.056 (2)	−0.0078 (18)
S1	0.0711 (5)	0.0404 (4)	0.0516 (5)	0.0027 (4)	0.0181 (5)	0.0008 (4)

### Geometric parameters (Å, °)

**Table:**

C1—O1	1.461 (4)	C17—H17A	0.9600
C1—H1A	0.9600	C17—H17B	0.9600
C1—H1B	0.9600	C17—H17C	0.9600
C1—H1C	0.9600	C18—O5	1.188 (3)
C2—O2	1.178 (4)	C18—O6	1.299 (4)
C2—O1	1.306 (4)	C18—C19	1.509 (4)
C2—C3	1.524 (4)	C19—N2	1.446 (3)
C3—N1	1.448 (3)	C19—C20	1.533 (4)
C3—C4	1.535 (4)	C19—H19	0.9800
C3—H3	0.9800	C20—C21	1.491 (4)
C4—C5	1.506 (4)	C20—H20B	0.9700
C4—H4A	0.9700	C20—H20A	0.9700
C4—H4B	0.9700	C21—C26	1.377 (4)
C5—C10	1.371 (4)	C21—C22	1.378 (4)
C5—C6	1.382 (4)	C22—C23	1.374 (5)
C6—C7	1.373 (5)	C22—H22	0.9300
C6—H6	0.9300	C23—C24	1.357 (6)
C7—C8	1.350 (5)	C23—H23	0.9300
C7—H7	0.9300	C24—C25	1.374 (6)
C8—C9	1.357 (5)	C24—H24	0.9300
C8—H8	0.9300	C25—C26	1.373 (5)
C9—C10	1.383 (5)	C25—H25	0.9300
C9—H9	0.9300	C26—H26	0.9300
C10—H10	0.9300	C27—C28	1.417 (7)
C11—O3	1.229 (3)	C27—O7	1.445 (6)
C11—N1	1.327 (3)	C27—H27A	0.9700
C11—C12	1.465 (4)	C27—H27B	0.9700
C12—C13	1.345 (4)	C28—C29	1.430 (7)
C12—S1	1.711 (3)	C28—H28B	0.9700
C13—C14	1.414 (4)	C28—H28A	0.9700
C13—H13	0.9300	C29—C30	1.489 (6)
C14—C15	1.342 (4)	C29—H29B	0.9700
C14—H14	0.9300	C29—H29A	0.9700
C15—C16	1.491 (4)	C30—O7	1.333 (4)
C15—S1	1.716 (3)	C30—H30A	0.9700
C16—O4	1.232 (3)	C30—H30B	0.9700
C16—N2	1.328 (3)	N1—H1	0.8600
C17—O6	1.465 (4)	N2—H2	0.8600
			
O1—C1—H1A	109.5	O6—C18—C19	113.8 (3)
O1—C1—H1B	109.5	N2—C19—C18	114.4 (3)
H1A—C1—H1B	109.5	N2—C19—C20	110.9 (2)
O1—C1—H1C	109.5	C18—C19—C20	110.7 (2)
H1A—C1—H1C	109.5	N2—C19—H19	106.8
H1B—C1—H1C	109.5	C18—C19—H19	106.8
O2—C2—O1	123.1 (3)	C20—C19—H19	106.8
O2—C2—C3	124.7 (3)	C21—C20—C19	112.6 (2)
O1—C2—C3	112.2 (3)	C21—C20—H20B	109.1
N1—C3—C2	109.7 (3)	C19—C20—H20B	109.1
N1—C3—C4	113.1 (2)	C21—C20—H20A	109.1
C2—C3—C4	110.3 (2)	C19—C20—H20A	109.1
N1—C3—H3	107.9	H20B—C20—H20A	107.8
C2—C3—H3	107.9	C26—C21—C22	117.8 (3)
C4—C3—H3	107.9	C26—C21—C20	120.9 (3)
C5—C4—C3	112.8 (2)	C22—C21—C20	121.3 (3)
C5—C4—H4A	109.0	C23—C22—C21	121.3 (4)
C3—C4—H4A	109.0	C23—C22—H22	119.3
C5—C4—H4B	109.0	C21—C22—H22	119.3
C3—C4—H4B	109.0	C24—C23—C22	120.8 (4)
H4A—C4—H4B	107.8	C24—C23—H23	119.6
C10—C5—C6	117.9 (3)	C22—C23—H23	119.6
C10—C5—C4	121.7 (3)	C23—C24—C25	118.3 (4)
C6—C5—C4	120.5 (3)	C23—C24—H24	120.8
C7—C6—C5	121.2 (3)	C25—C24—H24	120.8
C7—C6—H6	119.4	C26—C25—C24	121.5 (5)
C5—C6—H6	119.4	C26—C25—H25	119.3
C8—C7—C6	119.8 (4)	C24—C25—H25	119.3
C8—C7—H7	120.1	C25—C26—C21	120.3 (4)
C6—C7—H7	120.1	C25—C26—H26	119.8
C7—C8—C9	120.5 (4)	C21—C26—H26	119.8
C7—C8—H8	119.7	C28—C27—O7	105.8 (5)
C9—C8—H8	119.7	C28—C27—H27A	110.6
C8—C9—C10	119.9 (4)	O7—C27—H27A	110.6
C8—C9—H9	120.0	C28—C27—H27B	110.6
C10—C9—H9	120.0	O7—C27—H27B	110.6
C5—C10—C9	120.7 (3)	H27A—C27—H27B	108.7
C5—C10—H10	119.7	C27—C28—C29	107.3 (5)
C9—C10—H10	119.7	C27—C28—H28B	110.3
O3—C11—N1	121.2 (3)	C29—C28—H28B	110.3
O3—C11—C12	119.3 (3)	C27—C28—H28A	110.3
N1—C11—C12	119.5 (3)	C29—C28—H28A	110.3
C13—C12—C11	131.8 (3)	H28B—C28—H28A	108.5
C13—C12—S1	111.4 (2)	C28—C29—C30	105.0 (5)
C11—C12—S1	116.8 (2)	C28—C29—H29B	110.8
C12—C13—C14	112.7 (3)	C30—C29—H29B	110.8
C12—C13—H13	123.7	C28—C29—H29A	110.8
C14—C13—H13	123.7	C30—C29—H29A	110.8
C15—C14—C13	113.1 (3)	H29B—C29—H29A	108.8
C15—C14—H14	123.4	O7—C30—C29	105.7 (4)
C13—C14—H14	123.4	O7—C30—H30A	110.6
C14—C15—C16	130.6 (3)	C29—C30—H30A	110.6
C14—C15—S1	111.1 (2)	O7—C30—H30B	110.6
C16—C15—S1	118.1 (2)	C29—C30—H30B	110.6
O4—C16—N2	123.9 (3)	H30A—C30—H30B	108.7
O4—C16—C15	121.2 (3)	C11—N1—C3	120.8 (2)
N2—C16—C15	114.8 (2)	C11—N1—H1	119.6
O6—C17—H17A	109.5	C3—N1—H1	119.6
O6—C17—H17B	109.5	C16—N2—C19	123.0 (2)
H17A—C17—H17B	109.5	C16—N2—H2	118.5
O6—C17—H17C	109.5	C19—N2—H2	118.5
H17A—C17—H17C	109.5	C2—O1—C1	116.5 (3)
H17B—C17—H17C	109.5	C18—O6—C17	116.6 (3)
O5—C18—O6	124.1 (3)	C30—O7—C27	107.6 (4)
O5—C18—C19	122.1 (3)	C12—S1—C15	91.70 (14)
			
O2—C2—C3—N1	−27.1 (5)	N2—C19—C20—C21	−61.3 (3)
O1—C2—C3—N1	152.6 (3)	C18—C19—C20—C21	170.6 (3)
O2—C2—C3—C4	98.1 (4)	C19—C20—C21—C26	93.0 (4)
O1—C2—C3—C4	−82.3 (3)	C19—C20—C21—C22	−85.4 (4)
N1—C3—C4—C5	−63.2 (3)	C26—C21—C22—C23	0.3 (5)
C2—C3—C4—C5	173.6 (3)	C20—C21—C22—C23	178.8 (3)
C3—C4—C5—C10	100.2 (3)	C21—C22—C23—C24	−0.4 (6)
C3—C4—C5—C6	−79.2 (4)	C22—C23—C24—C25	0.4 (7)
C10—C5—C6—C7	0.7 (5)	C23—C24—C25—C26	−0.2 (8)
C4—C5—C6—C7	−179.9 (3)	C24—C25—C26—C21	0.0 (7)
C5—C6—C7—C8	−2.0 (6)	C22—C21—C26—C25	−0.1 (5)
C6—C7—C8—C9	2.7 (6)	C20—C21—C26—C25	−178.6 (4)
C7—C8—C9—C10	−2.1 (6)	O7—C27—C28—C29	−11.0 (9)
C6—C5—C10—C9	−0.1 (5)	C27—C28—C29—C30	−6.4 (9)
C4—C5—C10—C9	−179.6 (3)	C28—C29—C30—O7	23.0 (7)
C8—C9—C10—C5	0.9 (5)	O3—C11—N1—C3	−8.9 (4)
O3—C11—C12—C13	−173.0 (3)	C12—C11—N1—C3	170.4 (2)
N1—C11—C12—C13	7.7 (5)	C2—C3—N1—C11	−87.8 (3)
O3—C11—C12—S1	8.6 (4)	C4—C3—N1—C11	148.6 (3)
N1—C11—C12—S1	−170.8 (2)	O4—C16—N2—C19	9.1 (4)
C11—C12—C13—C14	−178.4 (3)	C15—C16—N2—C19	−170.2 (2)
S1—C12—C13—C14	0.1 (3)	C18—C19—N2—C16	−107.0 (3)
C12—C13—C14—C15	−0.8 (4)	C20—C19—N2—C16	127.0 (3)
C13—C14—C15—C16	176.3 (3)	O2—C2—O1—C1	−0.3 (5)
C13—C14—C15—S1	1.2 (3)	C3—C2—O1—C1	−179.9 (3)
C14—C15—C16—O4	−159.8 (3)	O5—C18—O6—C17	−0.1 (5)
S1—C15—C16—O4	15.0 (3)	C19—C18—O6—C17	−178.2 (3)
C14—C15—C16—N2	19.5 (4)	C29—C30—O7—C27	−30.3 (6)
S1—C15—C16—N2	−165.68 (19)	C28—C27—O7—C30	26.6 (7)
O5—C18—C19—N2	173.3 (3)	C13—C12—S1—C15	0.5 (2)
O6—C18—C19—N2	−8.6 (4)	C11—C12—S1—C15	179.2 (2)
O5—C18—C19—C20	−60.6 (4)	C14—C15—S1—C12	−1.0 (2)
O6—C18—C19—C20	117.5 (3)	C16—C15—S1—C12	−176.8 (2)

### Hydrogen-bond geometry (Å, °)

**Table:**

*D*—H···*A*	*D*—H	H···*A*	*D*···*A*	*D*—H···*A*
N2—H2···O7	0.86	2.02	2.859 (3)	164
